# MT-SCnet: multi-scale token divided and spatial-channel fusion transformer network for microscopic hyperspectral image segmentation

**DOI:** 10.3389/fonc.2024.1469293

**Published:** 2024-12-03

**Authors:** Xueying Cao, Hongmin Gao, Haoyan Zhang, Shuyu Fei, Peipei Xu, Zhijian Wang

**Affiliations:** ^1^ College of Computer Science and Software Engineering, Hohai University, Nanjing, China; ^2^ Department of Hematology, Nanjing Drum Tower Hospital Clinical College of Nanjing University of Chinese Medicine, Nanjing, China; ^3^ Department of Hematology, Nanjing University Medical School Affiliated Nanjing Drum Tower Hospital, Nanjing, China

**Keywords:** microscopic hyperspectral image, feature fusion, multi-scale, transformer, deformable convolution

## Abstract

**Introduction:**

Hybrid architectures based on convolutional neural networks and Transformers, effectively captures both the local details and the overall structural context of lesion tissues and cells, achieving highly competitive segmentation results in microscopic hyperspectral image (MHSI) segmentation tasks. However, the fixed tokenization schemes and single-dimensional feature extraction and fusion in existing methods lead to insufficient global feature extraction in hyperspectral pathology images.

**Methods:**

Base on this, we propose a multi-scale token divided and spatial-channel fusion transformer network (MT-SCnet) for MHSIs segmentation. Specifically, we first designed a Multi-Scale Token Divided module. It divides token at different scale based on mirror padding and promotes information interaction and fusion between different tokens to obtain more representative features for subsequent global feature extraction. Secondly, a novel spatial channel fusion transformer was designed to capture richer features from spatial and channel dimensions, and eliminates the semantic gap between features from different dimensions based on cross-attention fusion block. Additionally, to better restore spatial information, deformable convolutions were introduced in decoder.

**Results:**

The Experiments on two MHSI datasets demonstrate that MT-SCnet outperforms the comparison methods.

**Discussion:**

This advance has significant implications for the field of MHSIs segmentation. Our code is freely available at https://github.com/sharycao/MT-SCnet.

## 1 Introduction

The outstanding performance of hyperspectral imaging technology in remote sensing has attracted attention from various domains (1–3). Some researchers applied hyperspectral imaging to medical field, obtaining microscopic hyperspectral images (MHSIs) (4, 5). Compared to other images, MHSIs contain not only in high spatial resolution but also in high spectral resolution. These abundant spectral features can reflect the biochemical status of biological tissue cells, providing multi-dimensional information for tissue analysis and diagnosis (6). This provides strong support for the early diagnosis and treatment of diseases. The MHSIs segmentation task is the initial step in utilizing MHSIs to assist pathologists in diagnosis. Fully and effectively employing spectral-spatial information to segment tissues not only has significant research value, but also has critical clinical importance.

In early segmentation tasks, researchers focused on extracting spectral features (7–9) to segment the tissue. To further enhance the performance, some researchers employed methods such as Otsu (10), object-based multiscale analysis (11), and spatial-spectral density analysis (12) to both extract spatial and spectral features in MHSI. With the development of deep learning, the encoder-decoder architectures based on convolutional neural networks (CNNs) (13) have been widely applied in MHSI segmentation tasks. It can automatically extract spectral-spatial information from MHSIs, avoiding the need for complex manually designed features. For example, Sun et al. (14) designed a cholangiocarcinoma analysis and diagnosis method based on CNNs and proposed a spectral interval convolution and normalization scheme to learn richer spatial-spectral information. Wang et al. (15) designed a 3D fully convolutional network to extract spatial-spectral features from MHSIs to segment melanoma. Gao et al. (16) designed a high-level feature channel attention U-Net. Given the exceptional representation learning ability of CNNs, these methods have produced remarkable results. However, due to the limit by the inherent locality of CNNs, they cannot extract long-range context and global semantics features (17).

Vision Transformer (ViT) (18) is a structure based on self-attention that possesses powerful capabilities for global context modeling and has achieved excellent performance in various tasks. Naturally, incorporating it into MHSI segmentation has also become the key point of current research. Dai et al. (19) proposed a segmentation network based on swin-spec transformer to extract feature from both spatial and spectral dimensions of cholangiocarcinoma hyperspectral images. Wang et al. (20) designed a dual-modal pathological image cross-attention U-Net, which designed two cascaded multi head self-attention for extracting and exchanging the information between HSI and RGB. The incorporation of global context has contributed to the outstanding performance of these methods. However, these methods typically tokenize based on specific kernel scales, resulting in fixed size area information within the tokens. This limitation restricts the efficient extraction of subsequent global feature. Additionally, most existing methods extract features only from the spatial dimension, which result in insufficient feature extraction. Although some researchers extract features from both spectral and spatial dimensions, they often overlook the potential semantic gap between features from different dimensions. This may introduce new interference, thereby adversely affecting the model’s performance.

To address the aforementioned issues, this paper proposes a novel network called multi-scale token division and spatial-channel fusion transformer (MT-SCnet) for MHSIs segmentation. MT-SCnet aims to more efficiently extract the spatial-spectral information in MHSI, which mainly featuring on Multi-scale Token Division (MSTD) module and Spatial-Channel Fusion Transformer (SCFormer) block. Specifically, MSTD is designed to exploit the advantages of multi-scale tokens to enrich the global dependencies. It utilizes mirror flipping padding to generate different spatial size feature maps and divides tokens at different scale on them. Meanwhile, it promotes information interaction and fusion between tokens for richer and more robust feature information. SCFormer is proposed to more comprehensively exploit global spectral-spatial information. It extracts spectral-spatial information from both spatial and channel dimensions to obtain more enriched feature representations, and suppress the semantic gap between features from different dimensions through a cross attention fusion module (CAF). Furthermore, dense connection is introduced between channel dimensions to facilitate the transfer and interaction of global features across different levels. In addition, for capture local spectral-spatial information, MT-SCnet employs CNN at shadow encoder, and employ deformable convolutions (21) to better restore the spatial dimensions of the feature maps at decoder. The main contributions are as follows:

1. We propose MT-SCnet for MHSI segmentation, which more effectively and efficiently captures the spectral-spatial information in MHSIs through multi-scale token division and multi-dimensional feature extraction. The proposed MSTD, SCFormer, and the deformable convolutions all play crucial roles in the network, which enhanced its overall segmentation performance.
2. We propose a multi-scale token division module, which enrich the global dependencies by capturing multi-scale tokens and promoting fusion between different tokens.
3. We design a novel spatial-channel fusion transformer block. It conducts a more comprehensive extraction of global feature, and reduce the semantic gap between different dimensions features through emphasizes the commonality among them.
4. The experimental results on Gastric Intraepithelial Ieoplasia (GIN) and intestinal metaplasia (IM) MHSI datasets demonstrate that the proposed method achieves competitive results.

## 2 Related work

### 2.1 CNN for medical segmentation

The encoder-decoder architectures based on CNNs, such as U-Net (22) and U-Net++ (23) have exhibited remarkable performance in medical image segmentation tasks. Currently, researchers have conducted thorough investigations on the encoder-decoder architectures and proposed many methods to improve segmentation performance. For instance, some studies introducing residual structures into U-Net to address the issue of network degradation (24, 25). To obtain a better receptive field and capture more contextual information, researchers have introduced dilated convolutions (26) and deformable convolutions (27) to U-Net. Additionally, many studies have utilized attention mechanisms to help model focus on crucial feature information, which can further improve segmentation accuracy (28). For example, Gao et al. (29) designed an attention network for the segmentation of cholangiocarcinoma MHSIs. Liu et al. (30) designed the global context and hybrid attention network for lung segmentation.

### 2.2 Vision Transformer for medical segmentation

Since the outstanding achievements of ViT in 2020, the numerous of transformer-based methods have emerged in CV tasks. These methods divide the input image into patches and treat each patch as a token, which processed through transformer layers that includes self-attention mechanisms and feed-forward networks. Considering the high computational cost of directly processing the entire feature map, Swin-Transformer (31) divides the input image into non-overlapping windows and applies attention mechanisms independently within each window to reduce computational costs. Base on this, SwinUnet (32) was proposed and has demonstrated outstanding results in medical image segmentation.

Compared with using pure Transformer, some researchers designed hybrid networks that concurrently utilize CNNs and Transformers to achieve higher segmentation performance. TransUnet (17) was the first to design a hybrid architecture based on CNN and Transformer, and achieved excellent segmentation results. Huang et al. (33) introduced MISSFormer, a network designed to capture more discriminative dependencies and context, and has better ability to integrate global information and local context. To further minimize feature loss during the downsampling process and enhance the restoration of spatial information during upsampling, Zhang et al. (34) proposed the FDR-TransUNet based on TransUnet. This module introduces an amalgamation of concepts from densenet and resnet in encoder, and upsample through two independent expanding paths. Zhu et al. (35) proposed a parallel hybrid architecture that feeds input images concurrently into both CNN and transformer branches, thereby effectively merging spatial detail features with global contextual information.

### 2.3 Multi-scale information extraction for medical segmentation

To address the complex structural variations in biological tissues, multi-scale features are commonly utilized in medical image segmentation tasks. To enhance the quality of feature learning, Lin et al. (36) proposed a dual-branch Swin-transformer in the encoder to extract multi-scale feature representations. He et al. (37) developed a block within the encoding pathway that integrates multi-scale information, global features from transformers, and local details from CNNs, thereby enhancing the model’s capability for feature representation. Ao et al. (38) designed a shunt transformer to capture multi-scale features and utilized a pyramid decoder for decoding, effectively harnessing the fine features. In addition, some researchers employ multi-scale feature extraction strategies to bridge the gap between the features in the encoder and decoder, thereby enhancing the segmentation accuracy. For example, Fang et al. (39) designed a pyramid input-output network to compress multi-scale features, which for reducing the semantic gap between multi-scale features. Sun et al. (40) developed a multi-scale bridging module between the encoder and decoder to effectively interact with multi-scale context information. Liu et al. (41) proposed a multi-scale embedding spatial transformer. This module effectively captures the global context of images by modeling the spatial relationships between multi-scale and multi-level image patches. To further achieve a refined fusion of global and local features, Heidari et al. (42) designed multiple multi-scale representations based on the Swin Transformer and CNN-based encoders. Furthermore, some researchers have designed feature fusion structures within the decoder to fully decode features at various scales. Such as Yang et al. (43) proposed a multi query attention module to fuse the multi-scale features from different levels of decoder sub-network.

Although previous works have utilized CNNs and Transformers to extract global and local spectral-spatial information and further enhanced model representation through multi-scale feature extraction, their single tokenization schemes and simplistic fusion methods between features from different dimensions have limited the models’ performance potential.

## 3 Methods

**Figure 1:**
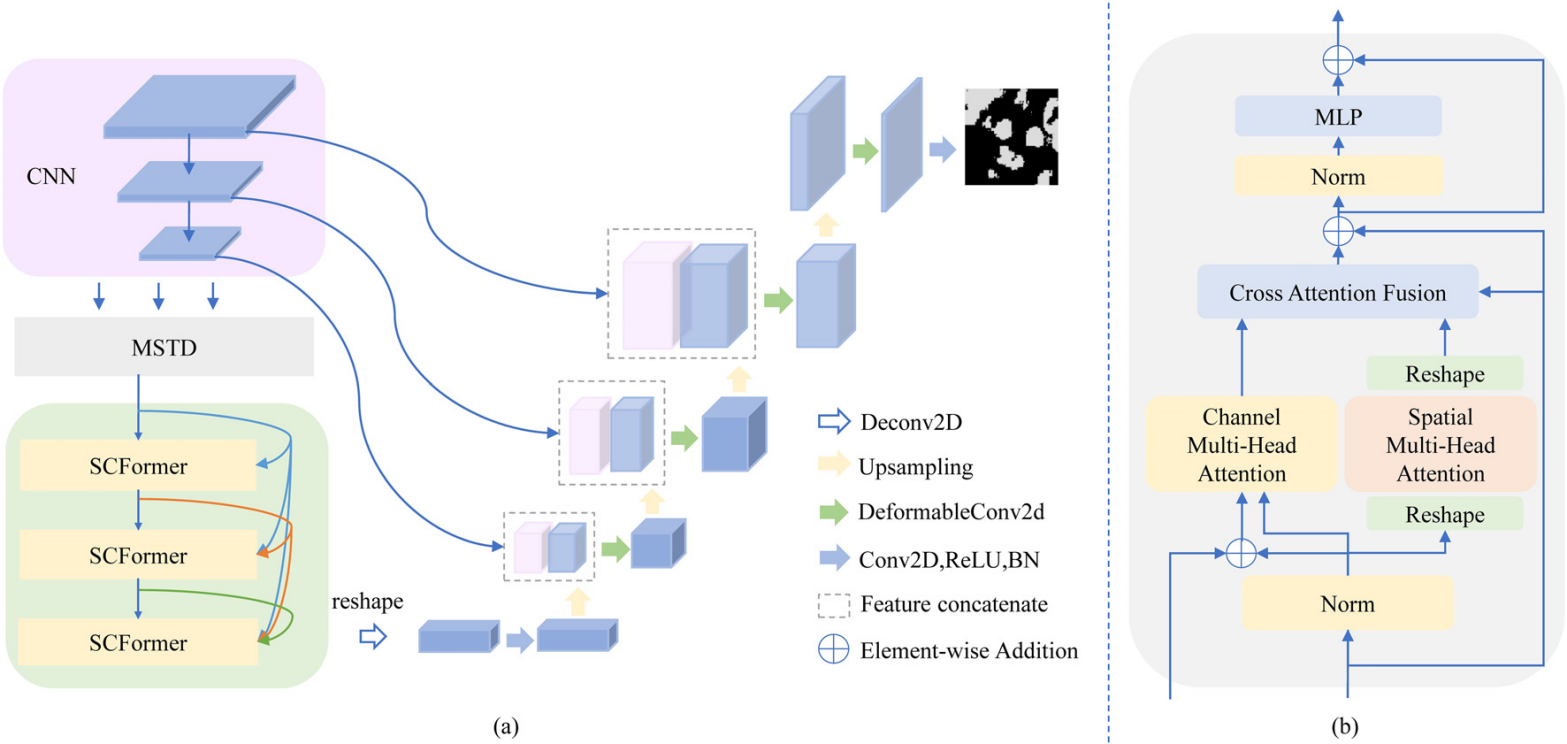
Architecture of the MT-SCnet. In **(A)**, we show the overall architecture of MT-SCnet. In **(B)**, we present details of the SCFormer.

As shown in **Figure 1**, MT-SCnet is an encoder-decoder network. The encoder consists of CNN, MSTD, and three SCFormers to extract local and global spectral-spatial information in MHSIs. In CNN, we adopted the design of TransUnet (17) to learn the local context in MHSI. Next, MSTD is used to learn multi-scale tokens to acquire richer and better feature representations for subsequent global feature extract. Additionally, three SCFormer layers are employed to learn global spectral-spatial information, and the dense connect is used for enhancing the utilization of global features. In decoder, deformable convolutions are introduced to obtaining more representative features, thereby better restore the spatial size. We also employed Principal Component Analysis (PCA) to reduce the dimensionality of MHSI, thus mitigating computational costs.

### 3.1 MSTD

Existing methods typically use a single scale tokenization scheme, which limits the efficiency of subsequent global information extraction. Multi-scale information provides an effective way to address significant morphological differences between tissues and enhance the model’s ability to represent details. Therefore, dividing tokens in a more flexible manner to capture multi-scale information, thereby providing richer and more robust features for subsequent global feature extraction, is key to improving segmentation performance. Based on this, MSTD is proposed to perform multi-scale token division and promote information interaction and fusion between tokens.

**Figure 2:**
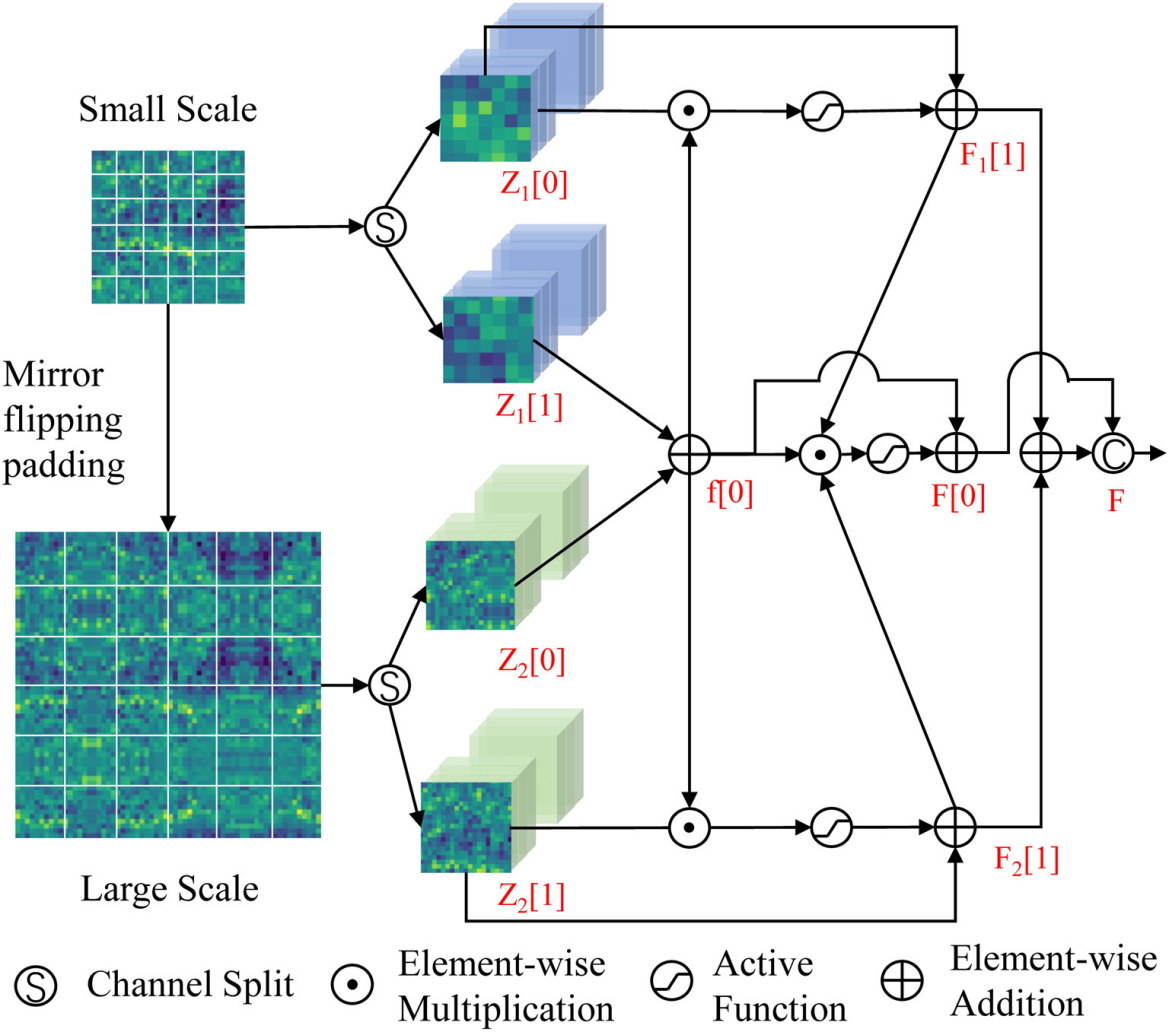
Structure of MSTD.


The specific steps of MSTD are shown in **Figure 2**. Assuming H and W  represent the height and width of the feature map Z  learned through CNN. Firstly, MSTD performs multi-scale token division based on convolution operations and mirror flipping. It uses the convolution of 2×2 kernels with 2 stride to obtain tokens $Z_{1}$ . Then, employ mirror flipping padding expand the Z to the size of 2 H ×2 W and use the convolution of 4×4 kernels with 4 stride to obtain tokens $Z_{2}$ . Although $Z_{1}$ and $Z_{2}$ have the same size and quantity, they contain different detailed information due to the different scale used for their division. Therefore, this step enables the obtain richer information. Secondly, MSTD promotes information interaction and fusion between tokens to generate more discriminative features. It evenly divides each scale tokens into two parts along the channel. This process is demonstrated in Equations 1, 2:

(1) $$(Z_{1}\left[0\right],Z_{1}\left[1\right])=split\left(Z_{1}\right)$$

(2) $$(Z_{2}\left[0\right],Z_{2}\left[1\right])=split\left(Z_{2}\right)$$

where split(·) represents the channel splitting operation. Then, $Z_{1}\left[1\right]$ and $Z_{2}\left[0\right]$ added together to obtain the fused feature f[0] . f[0] is element-wise multiplied with $Z_{1}\left[1\right]$ and $Z_{2}\left[1\right]$ respectively, to learn the weight matrix. The Tanh+1 was used as active function to map the aforementioned weight matrix to the range of [0,2]. In addition, residual connections are introduced to preserve the original information and accelerate convergence. The above process can be represented by Equations 3–5:


(3) $$f\left[0\right]=Z_{1}\left[1\right]+Z_{2}\left[0\right]$$

(4) $$F_{1}\left[1\right]=A\left(f\left[0\right]\times Z_{1}\left[0\right]\right)+Z_{1}\left[0\right]$$

(5) $$F_{2}\left[1\right]=A\left(f\left[0\right]\times Z_{2}\left[1\right]\right)+Z_{2}\left[1\right]$$

where A(·) represents Tanh+1 activation function, $F_{1}\left[1\right]$ and $F_{2}\left[1\right]$ represent the feature maps after residual connections. Then, $F_{1}\left[1\right]$ , $F_{2}\left[1\right]$ and f[0] are calculated through matrix element-wise multiplication to obatin the pixel-level relationship map. Tanh+1 and residual connections are also employed to enhance feature representation. The above operation enables $F_{1}\left[1\right]$ , $F_{2}\left[1\right]$ and f[0] incorporate feature information from different scales, thereby providing more abundant and robust feature information. Finally, the $F_{1}\left[1\right]$ and $F_{2}\left[1\right]$ are added together, and concatenate with F[0] along the channel dimension. Overall, dividing tokens at different scales and promoting information interaction and fusion between tokens of the same scale and different scales can provide richer and more comprehensive feature representations for subsequent global feature extraction. Assuming Concate{·} denotes the concatenate operation and F is the output of MSTD, the above process can be expressed as Equations 6, 7:


(6) $$F\left[0\right]=A\left(f\left[0\right]\times F_{1}\left[1\right]\times F_{2}\left[1\right]\right)+f\left[0\right]$$

(7) $$F=Concate\left\{F\left[0\right],\;\left(F_{1}\left[1\right]+F_{2}\left[1\right]\right)\right\}$$

### 3.2 SCFormer

**Figure 3:**
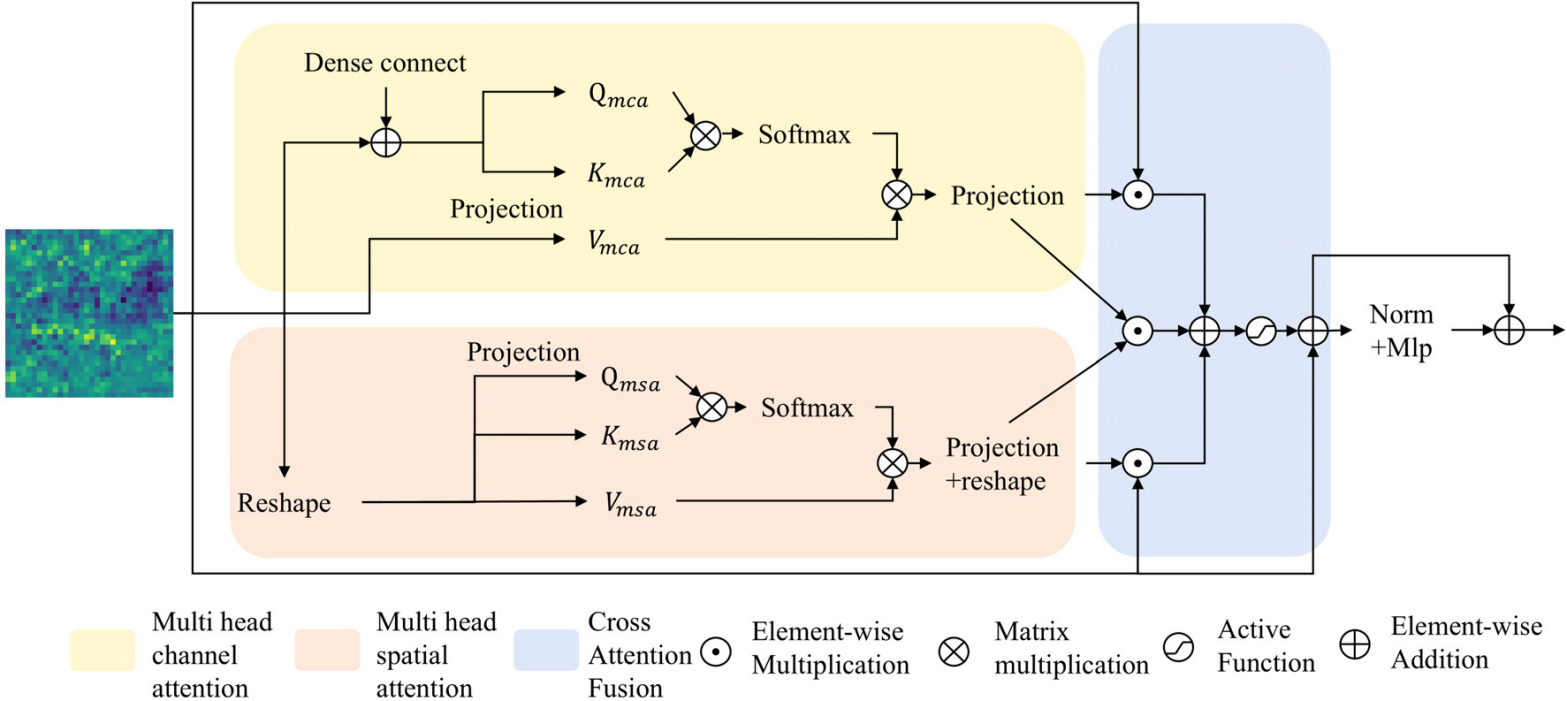
Structure of SCFormer.


Global features can better extract the context information of the overall structure of tissues, which is a crucial step for efficiently utilizing spectral-spatial information to learn tissue characteristics. However, most previous methods rely on learning from a single dimension, leading to insufficient extraction of global spectral-spatial information. Additionally, while some methods learn spectral-spatial information from different dimensions, they simply fuse these features. Considering the semantic gap between features from different dimensions, simple feature fusion may introduce new interference, thereby affecting the final segmentation results. Based on this, the SCFormer was proposed, which not only extract spectral-spatial information from spatial and channel dimensions by multi-head spatial attention (MSA) and multi-head channel attention (MCA), but also highlight common features between feature from different dimensions for suppressing the semantic gap by CAF. In addition, dense connections are incorporate between MCA to enhance feature reuse and improve the learning of features across different levels. As shown in **Figure 3**, the main improve structure of proposed SCFormer module incorporates MSA, MCA, CAF and multilayer perceptron (MLP). The MSA and MCA can be represented as Equations 8–13:

(8) $$MCA^{j}=softmax\left(\frac{Q_{mca}^{j}K_{mca}^{j}}{\sqrt{d_{k}}}\right)V_{mca}^{j}$$

(9) $$MSA^{j}=softmax\left(\frac{Q_{msa}^{j}K_{msa}^{j}}{\sqrt{d_{k}}}\right)V_{mca}^{j}$$

(10) $$Q_{mca}^{j}=LN\left(z^{\left(j-1\right)}+d\left(z\right)\right)$$

(11) $$K_{mca}^{j}=LN\left(z^{\left(j-1\right)}+d\left(z\right)\right)$$

(12) $$V_{mca}^{j}=LN\left(z^{\left(j-1\right)}\right)$$

(13) $$d\left(z\right)=LN\left(z^{\left(j-1\right)}+\ldots +z^{\left(1\right)}\right)$$

where $MCA^{j}$ and $MSA^{j}$ represents the MCA and MSA in the j -th SCFormer, respectively. $z^{\left(j-1\right)}$ represents the output feature map of the (j−1) -th SCFormer, d(·) represents the dense connection, and LN represents the fully connected layer.

For CAF in j -th SCFormer, we first perform element-wise multiplication between the j -th spatial feature and channel feature to highlight their commonalities, which pay more attention to the common important information while pay less attention on insignificance information. Subsequently, we perform same operation between the output of the (j−1) -th SCFormer with the j -th spatial feature and channel feature, respectively, to highlight the commonalities between different hierarchical features. Then, point-wise summation operation and Tanh+1 function is conduct. Overall, emphasizing the commonalities between features from different dimensions not only highlights common important features but also reduces redundancy and interference between features, thereby suppressing the semantic gap. It can generate the more efficiently fused feature map. Assuming $F_{out}^{j}$ as the output of j -th CAF, $F_{1}^{j}$ , $F_{2}^{j}$ and $F_{out}^{j-1}$ represent the output of $MCA^{j}$ , $MSA^{j}$ and (j−1) -th SCFormer respectively, the above process can be expressed as Equation 14:


(14) $$F_{out}^{j}=\;A\left(F_{1}^{j}\times F_{2}^{j}+F_{1}^{j}\times F_{out}^{j-1}+F_{2}^{j}\times F_{out}^{j-1}\right)$$

The MLP consists of two fully connected layers, GELU activation functions, and two dropout functions. Specific parameters can be referred to in TransUnet (17).

### 3.3 Decoder

In decoder, this paper introduces deformable convolution in decoder to adaptively adjust the shape of the receptive field. By flexibly choosing sampling locations to handle various deformations and scale variations, it better restores and refines the details in the feature maps. Compared to using multi-scale features or adding convolutional layers to enhance the decoder’s representation capability, deformable convolutions improve feature representation by introducing a small number of offset parameters. This approach enhances segmentation performance while avoiding a significant increase in computational cost. Deformable convolution can be represented as Equation 15:


(15) $$y=\sum_{k=1}^{K}w_{k}\times x\left(p+p_{k}+\Delta p_{k}\right)\times \Delta m_{k}$$

where K denote the sampling points of the convolution kernel. $p_{k}$ and $\Delta p_{k}$ represent the preset offset and the learnable offset, respectively. $w_{k}$ and $\Delta m_{k}$ correspond to the weight and the modulation scalar for the nth position, respectively.

### 3.4 Loss function

In the proposed model, the total training loss can be expressed as Equation 16:


(16) $$L_{seg}=0.7L_{ce}+0.3\frac{1}{C}\sum_{c=1}^{c}L_{DCE}^{c}$$

where $L_{ce}$ represents the Cross-Entropy Loss (CE), C denotes the number of classes, and $L_{DCE}^{c}$ represents the Dice Loss (DCE) for class C . CE measures the alignment between the predicted probabilities and the true labels, making it highly effective for optimizing pixel-level segmentation tasks. However, CE does not account for the spatial relationships between pixels and is sensitive to class imbalance issues. In contrast, DCE measures the overlap between the predicted and true regions, capturing the spatial relationships between pixels and highlighting target regions, thereby addressing the potential shortcomings of CE.

## 4 Experiments

### 4.1 Datasets

In order to validate the effectiveness of the proposed MT-SCnet, we conducted experiments on two MHSI datasets (44), including gastric mucosa intestinal metaplasia MHSI dataset (IM) and Gastric Intraepithelial Ieoplasia MHSI dataset (GIN). The dataset IM consists of 412 MHSIs and the dataset GIN consists of 282 MSHIs. Each hyperspectral data cube is acquired at 10x objective lens, which contains 40 bands and the spectral range of 450 to 700nm with 6.25nm spectral resolution. Under the guidance of pathologist, we select and crop the original MHSI images with 512×512 spatial resolution. All MHSIs are labelled by pathologists with precancerous regions. In this paper, five-fold cross validation method is used.

### 4.2 Implementation and evaluation

The MT-SCnet is implemented based on PyTorch, all experiments were conducted on a computer with 32GB of memory and an Nvidia GeForce GTX 4090. The stochastic gradient descent (SGD) is used for backpropagation, the learning rate was set to 0.01, momentum to 0.9, weight decay to 1e-6, and the batch size was 4. We utilized Dice Loss and Crossentropy functions simultaneously as loss functions, with weights set to 0.3 and 0.7, respectively. The number of epochs is set to 45 and 60 for dataset IM and GIN. The weights with the lowest loss were chosen as the optimal weights for testing. In order to better assess the performance of the proposed model, four common evaluation metrics are used: Overall Accuracy (OA), Sensitivity, Intersection Over Union (IoU) and Dice Similarity Coefficient (DSC).

### 4.3 Comparison with mainstream methods

We conducted a series of ablation experiments to validate the effectiveness of different modules in segmentation. We first tested the effectiveness of the MSTD, SCFormer and deformable convolution in MT-SCnet and further conducted ablation experiments within SCFormer and MSTD to validate the rationality of each design. All experiments were conducted on the IM dataset.

#### 4.3.1 IM dataset

As shown in **Table 1**, our model achieved the best result with 94.45% OA, 92.06% sensitivity, 86.63% IoU, and 92.82% DSC. It outperformed the purely CNN-based U-Net model by 1.26% OA, 1.46% Sensitivity, 2.75% IoU, and 1.59% DSC. Furthermore, compared to HLCA-Unet, which is also designed for MHSIs segmentation tasks, the proposed method achieves significant improvements across all metrics. In summary, MT-SCNet outperforms the pure CNN-based comparative models in terms of performance. Compared to the HiFormer-b, which utilizes both CNN and Transformer architectures and ranks second in segmentation accuracy, MT-SCnet showed improvements of 0.94% OA, 0.29% Sensitivity, 1.94% IoU and 1.11 DSC% respectively. This is benefited to the more efficiency learning of spectral-spatial information from MHSIs through MSTD, SCFormer and deformable convolutions. Additionally, MT-SCNet has 34.59M parameters and 120.91G FLOPs. Compared to HiFormer-b, which with 31.69M parameters and 355.77G FLOPs, the proposed method achieves a better balance in terms of accuracy, memory usage, and computational cost. The K, M, G, and T in the **Table 1** represent Kilo, Mega, Giga, and Tera, respectively.

**Table 1:** Comparison with other methods on IM dataset (%).

Architecture	OA	Sensitivity	IoU	DSC	Params	FLOPs
U-Net	93.19	90.60	83.88	91.23	34.53 M	1.05T
Att-Unet	93.01	90.46	85.19	91.00	34.88M	1.07T
HLCA-Unet	89.64	86.05	76.45	86.63	588.84K	62.92G
MISSFormer	92.44	90.37	82.41	90.33	35.45 M	36.96G
TransUnet	93.09	90.12	83.62	91.06	100.90 M	201.03G
Hiformer-b	93.51	91.77	84.69	91.71	31.69M	355.77G
MT-SCnet	94.45	92.06	86.63	92.82	34.59M	120.91G

The K, M, G, and T represent Kilo, Mega, Giga, and Tera, respectively.

**Figure 4:**
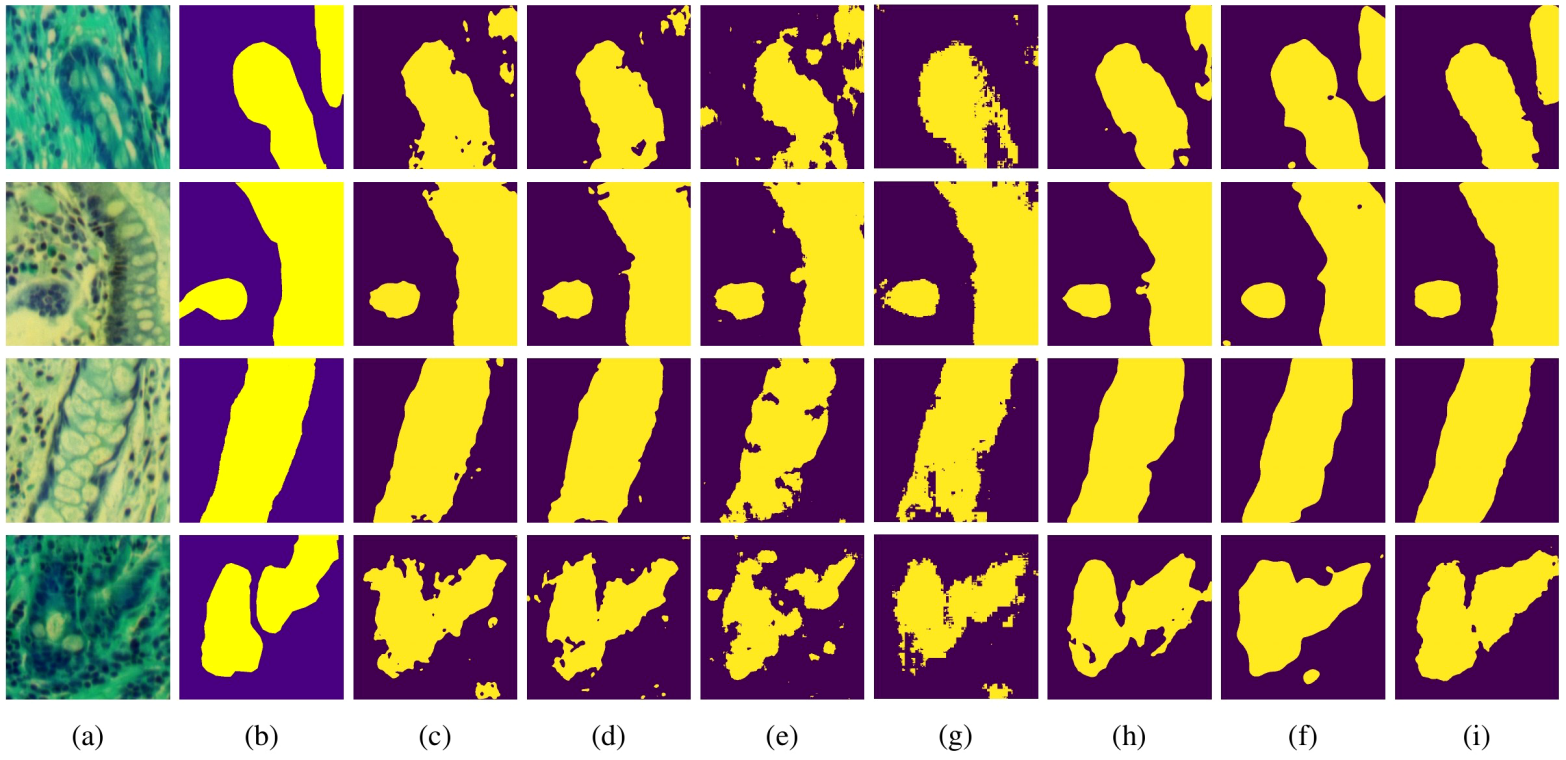
Visualization results of each model on MHSIs of IM: **(A)** false color image of hyperspectral images; **(B)** ground truth; **(C)** U-net; **(D)** Att-Unet; **(E)** HLCA-Unet; **(F)** MISSFormer; **(G)** TransUnet; **(H)** Hiformer-b; **(I)** MT-SCnet.

To further validate the segmentation performance of MT-SCnet on IM, the segmentation results of all models were visualized. As shown in **Figure 4**, the first column displays the false-color images of MHSIs, the second column shows the true labels, and the subsequent column present the prediction results of the U-Net, Att-Unet, HLCA-Unet, MISSFormer, TransUnet, HiFormer-b and MT-SCnet networks. As shown in **Figure 4**, the prediction maps of MT-SCNet exhibit smoother boundaries and provide a more accurate and comprehensive delineation of precancerous lesion areas. Compared to HiFormer-b, the proposed network more clearly delineates the boundaries of different tissues in densely distributed regions (as shown in the fourth row). In summary, MT-SCNet achieves superior recognition of contiguous regions, producing segmentation results that align more closely with the true labels compared to other models.

#### 4.3.2 GIN dataset

As presented in **Table 2**, MT-SCnet achieve the results with 88.93% OA, 88.58% sensitivity, 79.75% IoU, 88.71% DSC on the GIN dataset. Compared to other methods, the proposed method achieves the highest accuracy in terms of OA, IoU, and DSC. However, in terms of sensitivity, MT-SCNet performs slightly lower than U-Net and HiFormer-b. Considering that the cancerous regions predicted by MT-SCNet exhibit a higher overlap with the actual regions and demonstrate more accurate overall prediction accuracy, this may be attributed to the fact that, although U-Net and HiFormer-b are more sensitive in detecting cancerous regions, this sensitivity comes at the cost of generating more false positives. Overall, the proposed method demonstrates superior performance when considering all aspects comprehensively.

**Table 2:** Comparison with other methods on GIN dataset (%).

Architecture	OA	Sensitivity	IoU	DSC
U-net	88.44	88.95	79.11	88.33
Att-Unet	88.40	87.93	78.85	88.17
HLCA-Unet	85.08	82.26	73.06	84.42
MISSFormer	85.70	84.81	74.48	85.37
TransUnet	86.20	87.17	75.66	86.14
Hiformer-b	88.66	89.86	79.58	88.62
MT-SCnet	88.93	88.58	79.75	88.71

**Figure 5:**
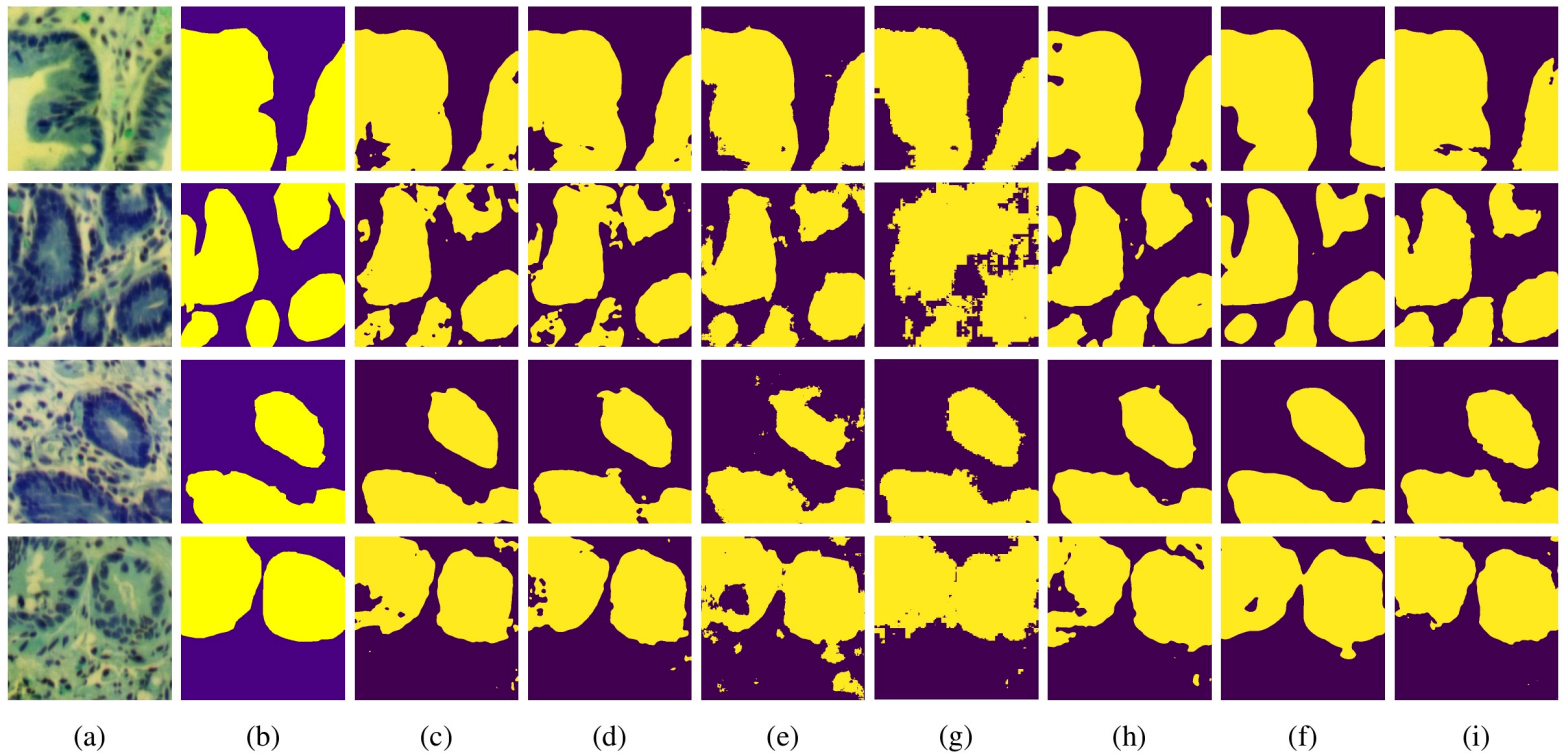
Visualization results of each model on MHSIs of GIN: **(A)** false color image of hyperspectral images; **(B)** ground truth; **(C)** U-net; **(D)** Att-Unet; **(E)** HLCA-Unet; **(F)** MISSFormer; **(G)** TransUnet; **(H)** Hiformer-b; **(I)** MT-SCnet.

We have also conducted visualization of the segmentation results on the GIN dataset. As shown in **Figure 5**, the first column shows the MHSI false color images, the second column represents the ground truth, and the subsequent columns sequentially display the comparison results of U-Net, Att-Unet, HLCA-Unet, MISSFormer, TransUnet, HiFormer-b and MT-SCnet. It is evident that, compared to other methods, MT-SCNet exhibits fewer misclassifications and omissions, demonstrating more accurate segmentation performance with results that are closer to the ground truth. From the aforementioned results, it can be observed that the MT-SCnet in this paper demonstrates stronger segmentation performance.

### 4.4 Ablation experiments

We conducted a series of ablation experiments to validate the effectiveness of different modules in segmentation. We first tested the effectiveness of the MSTD, SCFormer and deformable convolution in MT-SCnet and further conducted ablation experiments within SCFormer to validate the rationality of each design. All experiments were conducted on the IM dataset. The result show in **Tables 3**, **4**. In the tables, × indicates that the module was not used, while √ indicates that it was employed.

**Table 3:** Ablation study on the proposed components of the MT-SCnet with the IM dataset (%).

No.	MSTD	SCFormer	Deformable Convolution	OA	Sensitivity	IoU	DSC
1	×	×	×	92.41	90.07	82.26	90.26
2	✓	×	×	92.87	91.07	83.39	90.92
3	×	✓	×	93.31	91.73	84.26	91.45
4	×	×	✓	93.90	91.03	85.36	92.09
5	✓	✓	×	93.70	93.17	85.25	92.03
6	✓	✓	✓	94.45	92.06	86.63	92.82

× indicates that the module was not used, while √ indicates that it was employed.

**Table 4:** Ablation study on the SCFormer of the MT-SCnet with the IM dataset (%).

No.	MSA	MCA	Dense Connect	CAF	OA	Sensitivity	IoU	DSC
1	✓	×	×	×	92.41	90.07	82.26	90.26
2	×	✓	×	×	92.56	90.29	82.60	90.46
3	×	✓	✓	×	92.71	90.18	82.86	90.62
4	✓	✓	✓	×	92.39	90.28	82.25	90.25
5	✓	✓	✓	✓	93.31	91.73	84.26	91.45

× indicates that the module was not used, while √ indicates that it was employed.

#### 4.4.1 Ablation study on proposed components

**Table 3** shows the ablation experimental results of the proposed components. Experiment 1 is the baseline result, which only uses three transformer blocks with MSA. The comparisons between Experiment 1 and Experiment 2, as well as between Experiment 3 and Experiment 5, demonstrate that the addition of MSTD has improved OA, Sensitivity, IoU, and DSC indicators. This indicates that the addition of MSTD has led to an increase in overall prediction accuracy, and the overlap and similarity of the segmented regions are also enhanced. Additionally, comparing Experiment 1 with Experiment 3, as well as Experiment 2 with Experiment 5, it is evident that the integration of SCFormer results in a significant segmentation performance improvement. Furthermore, from the comparison in Experiment 1 and Experiment 4, as well as Experiment 5 and Experiment 6, deformable convolution further enhances the segmentation accuracy of the model. This is because the incorporation of deformable convolution enhances local detail capture and improves the fusion of deep and shallow features. In summary, compared to the baseline, the proposed model achieved improvements of 2.04% OA, 1.99% Sensitivity, 4.37% IoU, and 2.56% DSC, achieving the best performance. Overall, the proposed modules all have a beneficial impact on the model’s segmentation performance.

#### 4.4.2 Ablation study on SCFormer module

Next, we conducted ablation experiments on SCFormer, and the results are presented in **Table 4**. As mentioned above, SCFormer extracts features from both spatial and channel dimensions by MSA and MCA, and then fuses these features from different dimensions based on a CAF. To evaluate the impact of these components on segmentation performance, five experiments were conducted in this section. All experiments were performed on the baseline model. The comparison between Experiment 2 and Experiment 3 shows that the addition of dense connections improves the model’s performance. In Experiment 4, MSA was added on the basis of Experiment 3. However, the results showed a certain degree of decline in OA, Sensitivity, and DSC metrics. This may be because the semantic gap from different dimensions makes simple fusion introduce new redundancy and interference. In Experiment 5, the CAF module was added to Experiment 4 to alleviate the semantic differences between features from different dimensions, resulting in the best performance among all experiments. This demonstrates that CAF can suppress the semantic gap between features from different dimensions by highlighting commonalities between feature, thereby enhancing the model’s segmentation performance.

#### 4.4.3 Ablation study on MSTD module

Finally, we perform an ablation study on MSTD and present the results in **Table 5**. In **Table 5**, MSTD1 represents divide multiscale token based on mirror padding, and MSTD2 represents the interaction and fusion of information between different token. With the addition of MSTD1, the model achieved improvements of 0.06%, 0.11%, and 0.06% in OA, IoU, and DSC, respectively, while sensitivity decreased by 0.02%. After add the MSTD2, the model achieved 92.87% in OA, 91.07% in sensitivity, 83.39% in IoU, and 90.92% in DSC, which improve the increases of 0.4% in OA, 1.02% in sensitivity, 1.02% in IoU, and 0.6% in DSC compare with using only MSTD1. The above results indicate that using MSTD1 alone provides limited contributions to model accuracy, likely due to the consideration of only two scales during token partitioning, resulting in limited detail capture. However, with the addition of MSTD2, the proposed method shows substantial improvements across all four evaluation metrics. This confirms that promoting interaction and fusion between markers of different sizes to obtain more discriminative features can further improve segmentation performance.

**Table 5:** Ablation study results on the key blocks of the MSTD with the IM dataset (%).

No.	MSTD1	MSTD2	OA	Sensitivity	IoU	DSC
1	×	×	92.41	90.07	82.26	90.26
2	✓	×	92.47	90.05	82.37	90.32
3	✓	✓	92.87	91.07	83.39	90.92

× indicates that the module was not used, while √ indicates that it was employed.

### 4.5 The effect of the PCA

**Figure 6:**
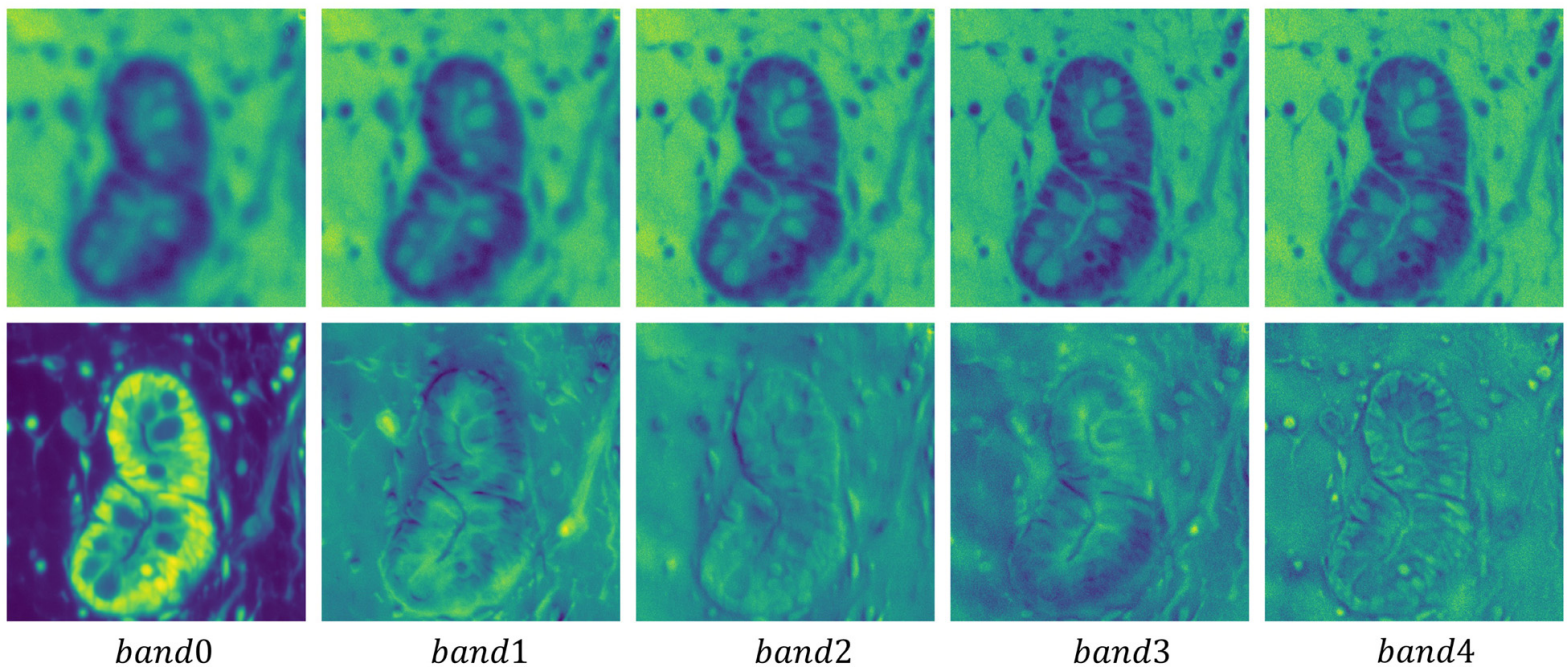
Visualization of the first five principal component of the original hyperspectral pathology image alongside the first five bands of the image after dimensionality reduction via PCA.

Due to the high correlation and similarity among spectral bands in hyperspectral pathology images, researchers typically employ PCA to preprocess these images. To further illustrate the rationale for using PCA, we present the first five principal component of the original MHSIs alongside the first five bands of the image after dimensionality reduction via PCA in **Figure 6**. The first row shows the original MHSIs, while the second row displays the images after PCA dimensionality reduction. It can be observed that the original spectral bands of the hyperspectral pathology image exhibit high similarity and a significant degree of correlation. After dimensionality reduction using PCA, the features between the principal components show significant variation, with the leading principal components retaining most of the useful information. This dimensionality reduction helps subsequent models focus on the most relevant feature information, thereby enhancing the efficiency and performance of image segmentation tasks.

**Figure 7:**
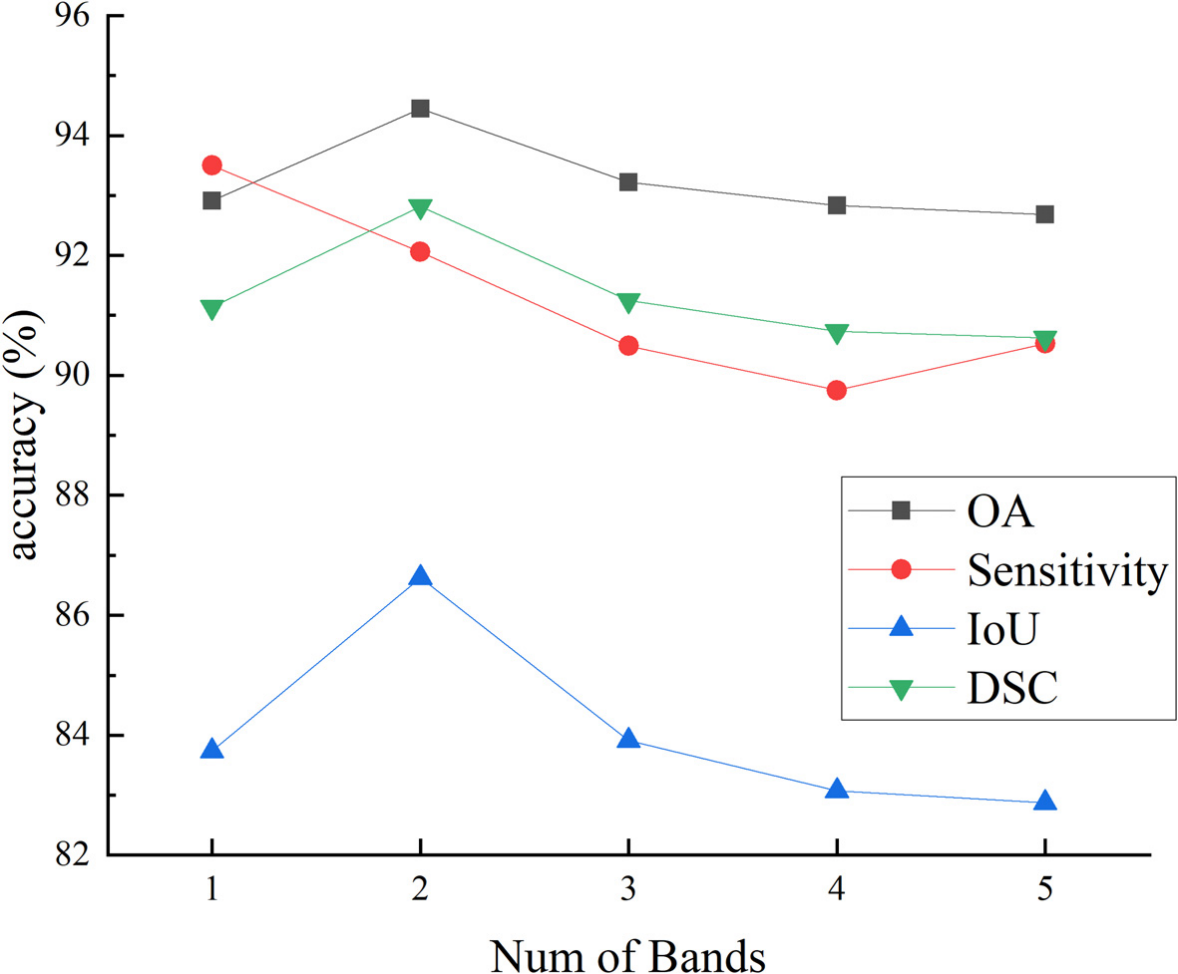
The effect of PCA on segmentation results on IM.

**Figure 8:**
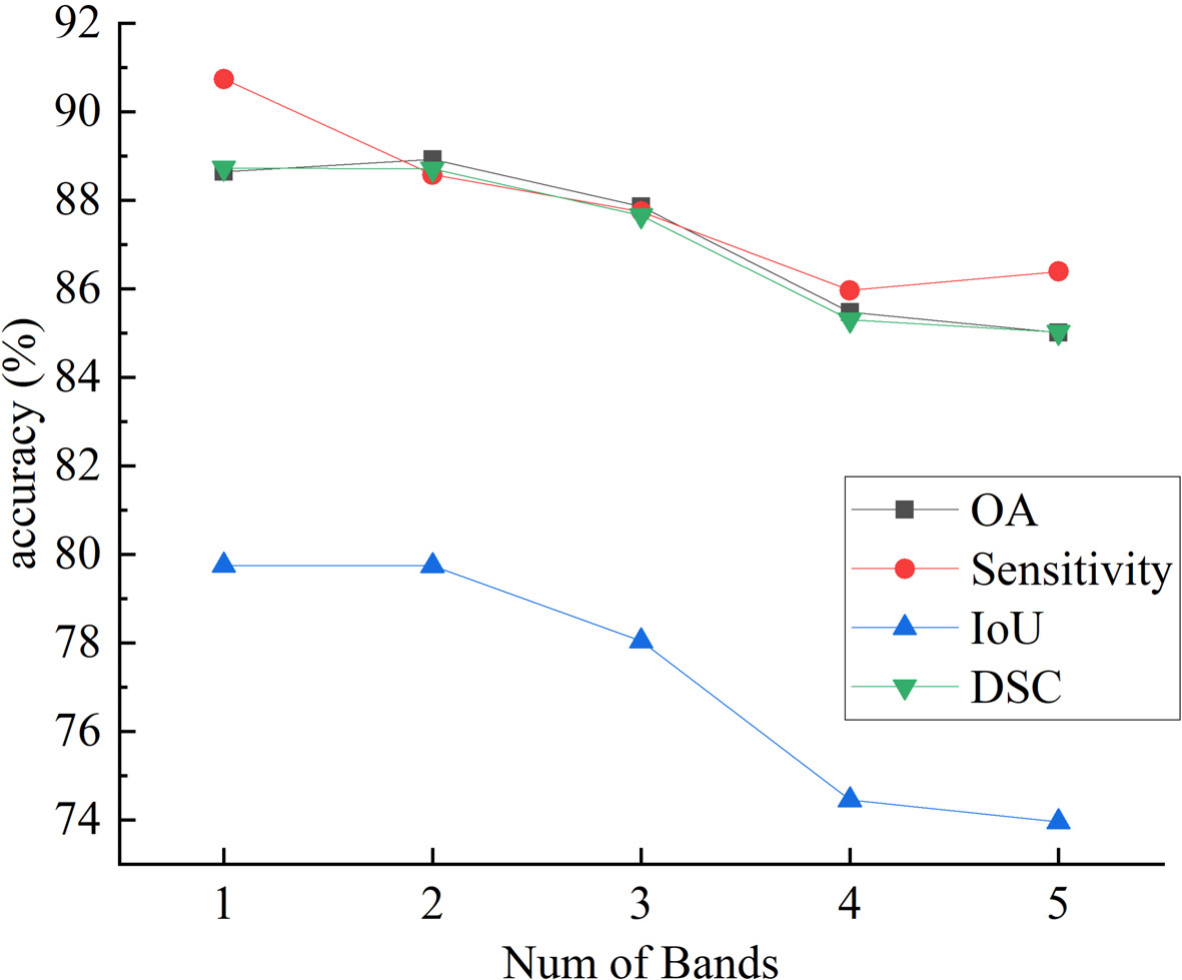
The effect of PCA on segmentation results on GIN.

**Figures 7** and **8** respectively illustrate the impact of PCA on the final segmentation results for the IM and GIN datasets. In the Figures, experiments 1 through 5 correspond to selecting the first 1 to 5 bands following PCA. The experiment results on two datasets demonstrate that as the number of bands increases, the performance on OA, IoU and DSC metrics initially improves and subsequently declines. This occurs because MHSI not only contain abundant spatial and spectral information but also includes redundant and interfering information. Increasing the number of bands excessively can introduce these features, consequently diminishing the segmentation accuracy. With the increase in the number of bands, the sensitivity results initially decrease significantly and then slightly increase. However, compared to using fewer PCA bands, the results still remain at a lower accuracy level. Overall, results on two datasets show that a moderate increase in the number of bands helps improve segmentation accuracy, but too many bands can lead to a decline in model segmentation performance.

### 4.6 The effect of token scale in MSTD

To study the impact of tokens at different scales on the final segmentation results, we evaluated the combinations that involved partitioning using kernels with (2,3), (2,4), (2,5), (3,5). For example, (2,3) means 2×2 kernel with 2 stride to generate 2×2 token, and use 3×3 kernel with 3 stride to generate 3×3 token. The results for IM and GIN are presented in **Tables 6**, **7**. The experiment results on the IM show that the best performance across all metrics occurs when the token scale combination is set to (2,4). For GIN, token scale set to (2,4) achieve the best results in OA, IoU and DSC, while the lowest results in Sensitivity. Additionally, the experimental results on the two datasets indicate that as the token size increases, the network segmentation performance generally shows a trend of initial improvement followed by a decline. In summary, we selected (2,4) as the final token partitioning dimensions in this study.

**Table 6:** Effect of division scale in MSTD on IM dataset (%).

No.	Dense Connect	OA	Sensitivity	IoU	DSC
1	(2,3)	94.36	91.34	86.35	92.66
2	(2,4)	94.45	92.06	86.63	92.82
3	(2,5)	94.08	91.01	85.68	92.27
4	(3,5)	93.37	89.74	84.07	91.33

**Table 7:** Effect of division scale in MSTD on GIN dataset (%).

No.	Dense Connect	OA	Sensitivity	IoU	DSC
1	(2,3)	87.32	90.79	77.96	87.58
2	(2,4)	88.93	88.58	79.75	88.71
3	(2,5)	88.61	90.56	79.66	88.67
4	(3,5)	88.42	89.45	79.18	88.36

## 5 Conclusion

In this study, we introduce a novel network named MT-SCnet for the segmentation of MHSIs. The most significant features of this network are its MSTD and SCFormer components. In MSTD, multi-scale token division and the interaction and fusion of information between different tokens provide richer and more robust feature information for subsequent global feature extraction. In SCFormer, global features are first extracted from both spatial and channel dimensions, and dense connections are introduced to obtain richer spectral-spatial information. Secondly, cross attention is used to highlight the common important features and reduce redundant information between different dimensions, thereby minimizing the semantic gap between features from different dimensions, further enhancing the model representation. Additionally, to better decode feature information in MHSIs, deformable convolutions are introduced. Results from two MHSIs datasets demonstrate that MT-SCnet exhibits strong performance, outperforming current state-of-the-art segmentation methods. In future studies, we will focus on exploring more flexible ways, such as quadrilateral, to divide tokens and on how to suppress the semantic gap between local context and global information.

## Data Availability

Requests to access these datasets should be directed to gaohongmin@hhu.edu.cn.

## References

[1] Chen Z Hong D Gao H. Grid network: Feature extraction in anisotropic perspective for hyperspectral image classification. IEEE Geosci Remote Sens Letters. (2023) 20:1–5. doi: 10.1109/LGRS.2023.3297612

[2] Zhang L Wang Y Yang L Chen J Liu Z Bian L. D^2^S^2^BoT: dual-dimension spectral-spatial bottleneck transformer for hyperspectral image classification. IEEE J Select Topics Appl Earthobservations Remote Sensing. (2024) 17:2655–69. doi: 10.1109/JSTARS.2023.3342461

[3] Liao D Shi C Wang L. A spectral-spatial fusion transformer network for hyperspectral image classification. IEEE Trans Geosci Remote Sensing. (2023) 61:1–16. doi: 10.1109/TGRS.2023.3286950

[4] Wang J Mao X Wang Y Tao X Chu J Li Q. Automatic generation of pathological benchmark dataset from hyperspectral images of double stained tissues. Optics Laser Technol. (2023) 163:109331. doi: 10.1016/j.optlastec.2023.109331

[5] Li Q He X Wang Y Liu H Xu D Guo F. Review of spectral imaging technology in biomedical engineering: achievements and challenges. J Biomed Optics. (2013) 18:100901. doi: 10.1117/1.JBO.18.10.100901 24114019

[6] Neittaanmaki-Perttu N Gronroos M Tani T Polonen I Ranki A Saksela O. Detecting field cancerization using a hyper-spectral imaging system. Lasers Surg Med. (2013) 45:410–7. doi: 10.1002/lsm.22160 24037822

[7] Lima C Correa L Byrne H Zezell D. K-means and Hierarchical Cluster Analysis as segmentation algorithms of FTIR hyperspectral images collected from cutaneous tissue. In: 2018 SBFoton International Optics and Photonics Conference (SBFoton IOPC). Campinas, Brazil: IEEE. (2018), pp. 1–4. doi: 10.1109/SBFoton-IOPC.2018.8610920

[8] Piqueras S Krafft C Beleites C Egodage K von Eggeling F Guntinas Lichius O. Combining multiset resolution and segmentation for hyperspectral image analysis of biological tissues. Anal Chimica Acta. (2015) 881:24–36. doi: 10.1016/j.aca.2015.04.053 26041517

[9] Li Q Wang Y Liu H Guan Y Xu L. Sublingual vein extraction algorithm based on hyperspectral tongue imaging technology. Computer Med Imaging Graph. (2011) 35:179–85. doi: 10.1016/j.compmedimag.2010.10.001 21030208

[10] Wang J Hu M Zhou M Sun L Li Q. Segmentation of pathological features of rat bile duct carcinoma from hyperspectral images. In: 2018 11th International Congress on Image and Signal Processing, BioMedical Engineering and Informatics (CISP-BMEI). Beijing, China: IEEE. (2018), pp. 1–5. doi: 10.1109/CISP-BMEI.2018.8633189

[11] Wang Q Li Q Zhou M Sun L Qiu S Wang Y. Melanoma and melanocyte identification from hyperspectral pathology images using object-based multiscale analysis. Appl Spectroscopy. (2018) 72:538–1547. doi: 10.1177/0003702818781352 29888955

[12] Lv M Li W Tao R Lovell NH Yang Y Tu T. Spatial-spectral density peaks-based discriminant analysis for membranous nephropathy classification using microscopic hyperspectral images. IEEE J Biomed Health Inform. (2021) 25:3041–51. doi: 10.1109/JBHI.2021.3050483 33434138

[13] Krizhevsky A Sutskever I Hinton GE. Imagenet classification with deep convolutional neural networks. Ommun ACM. (2017) 60:84–90. doi: 10.1145/3065386

[14] Sun L Zhou M Li Q Hu M Wen Y Zhang J. Diagnosis of cholangiocarcinoma from microscopic hyperspectral pathological dataset by deep convolution neural networks. Methods. (2022) 202:22–30. doi: 10.1016/j.ymeth.2021.04.005 33838272

[15] Wang Q Sun L Wang Y Zhou M Hu M Chen J. Identification of melanoma from hyperspectral pathology image using 3D convolutional networks. IEEE Trans Med Imag. (2021) 40:218–27. doi: 10.1109/TMI.2020.3024923 32956043

[16] Gao H Yang M Cao X Liu Q Xu P. A high-level feature channel attention UNet network for cholangiocarcinoma segmentation from microscopy hyperspectral images. Mach Vision Applications. (2023) 34:72. doi: 10.1007/s00138-023-01418-x

[17] Chen J Lu Y Yu Q Luo X Adeli E Wang Y. TransUnet: Transformers make strong encoders for medical image segmentation. arXiv. (2021). https://arxiv.org/abs/2102.04306.

[18] Dosovitskiy A Beyer L Kolesnikov A Weissenborn D Zhai X Unterthiner T. An image is worth 16x16 words: Transformers for image recognition at scale. arXiv. (2021). https://arxiv.org/abs/2010.11929.

[19] Dai K Zhou Z Qiu S Wang Y Zhou M Li M. A generative data augmentation trained by low-quality annotations for cholangiocarcinoma hyperspectral image segmentation. In: 2023 International Joint Conference on Neural Networks (IJCNN). Gold Coast, Australia: IEEE. (2023), pp. 01–9. doi: 10.1109/IJCNN54540.2023.10191749

[20] Wang J Zhang B Wang Y Zhou C Vonsky MS Mitrofanova LB. CrossU-Net: Dual-modality cross-attention u-net for segmentation of precancerous lesions in gastric cancer. Computer Med Imaging Graph. (2024) 112:102339. doi: 10.1016/j.compmedimag.2024.102339 38262134

[21] Zhu X Hu H Lin S Dai J. Deformable convnets V2: More deformable, better results. In: 2019 IEEE/CVF Conference on Computer Vision and Pattern Recognition (CVPR). Long Beach, CA, USA: IEEE. (2019). pp. 9300–8. doi: 10.1109/CVPR.2019.00953

[22] Ronneberger O Fischer P Brox T. U-net: Convolutional networks for biomedical image segmentation. arXiv. (2015). https://arxiv.org/abs/1505.04597.

[23] Zhou Z Siddiquee MMR Tajbakhsh N Liang J. Unet++: A nested u-net architecture for medical image segmentation. In: Deep Learning in Medical Image Analysis and Multimodal Learning for Clinical Decision Support: 4th International Workshop, DLMIA 2018, and 8th International Workshop, ML-CDS 2018, Held in Conjunction with MICCAI 2018. Granada, Spain. Berlin, Heidelberg: Springer-Verlag. (2018). pp. 3–11. doi: 10.1007/978-3-030-00889-5_1 PMC7329239 32613207

[24] Feng T Wang C Chen X Fan H Zeng K Li Z. URNet: A U-Net based residual network for image dehazing. Appl Soft Comput. (2021) 102:106884. doi: 10.1016/j.asoc.2020.106884

[25] Liu S Li Y Zhou J Hu J Chen N Shang Y. Segmenting nailfold capillaries using an improved U-net network. Microvascular Res. (2020) 130:104011. doi: 10.1016/j.mvr.2020.104011 32371104

[26] Xie X Pan X Zhang W An J. A context hierarchical integrated network for medical mage segmentation. Comput Electric Eng. (2022) 101:108029. doi: 10.1016/j.compeleceng.2022.108029

[27] Ghamsarian N Wolf S Zinkernagel M Schoeffmann K Sznitman R. DeepPyramid plus: medical image segmentation using pyramid view fusion and deformable pyramid reception. Int J Comput Assisted Radiol Surge. (2024) 19:851–9. doi: 10.1007/s11548-023-03046-2 PMC11585507 38189905

[28] Oktay O Schlemper J Folgoc LL Lee M Heinrich M Misawa K. Attention U-Net: Learning where to look for the pancreas. arXiv. (2018). https://arxiv.org/abs/1804.03999.

[29] Gao E Jiang H Zhou Z Yang C Chen M Zhu W. Automatic multi-tissue segmentation in pancreatic pathological images with selected multi-scale attention network. Comput Biol Med. (2022) 151:106228. doi: 10.1016/j.compbiomed.2022.106228 36306579

[30] Liu H Fu Y Zhang S Liu J Wang Y Wang G. GCHA-Net: Global context and hybrid attention network for automatic liver segmentation. Comput Biol Med. (2023) 152:106352. doi: 10.1016/j.compbiomed.2022.106352 36481761

[31] Liu Z Lin Y Cao Y Hu H Wei Y Zhang Z. Swin transformer: Hierarchical vision transformer using shifted windows. arXiv. (2021). https://arxiv.org/abs/2103.14030.

[32] Cao H Wang Y Chen J Jiang D Zhang X Tian Q. Swin-Unet: Unet-like pure transformer for medical image segmentation. arXiv. (2021). https://arxiv.org/abs/2105.05537.

[33] Huang X Deng Z Li D Yuan X Fu Y. MISSFormer: An effective transformer for 2D medical image segmentation. IEEE Trans Med Imag. (2023) 42:1484–94. doi: 10.1109/TMI.2022.3230943 37015444

[34] Zhang C Sun S Hu W Zhao P. FDR-TransUnet: A novel encoder-decoder architecture with vision transformer for improved medical image segmentation. Comput In Biol Med. (2023) 169:107858. doi: 10.1016/j.compbiomed.2023.107858 38113680

[35] Zhu J Sheng Y Cui H Ma J Wang J Xi H. Cross pyramid transformer makes U-Net stronger in medical image segmentation. Biomed Signal Process Control. (2023) 86:105361. doi: 10.1016/j.bspc.2023.105361

[36] Lin A Chen B Xu J Zhang Z Lu G Zhang D. DS-TransUNet: Dual swin transformer u-net for medical image segmentation. IEEE Trans Instrument Measure. (2022) 71:1–15. doi: 10.1109/TIM.2022.3178991

[37] He A Wang K Li T Du C Xia S Fu H. H2Former: An efficient hierarchical hybrid transformer or medical image segmentation. IEEE Trans Med Imag. (2023) 42:2763–75. doi: 10.1109/TMI.2023.3264513 37018111

[38] Ao Y Shi W Ji B Miao Y He W Jiang Z. MS-TCNet: An effective transformer-cnn combined network using multi-scale feature learning for 3d medical image segmentation. Comput Biol Med. (2024) 170:108057. doi: 10.1016/j.compbiomed.2024.108057 38301516

[39] Fang X Yan P. Multi-organ segmentation over partially labeled datasets with multi-scale feature abstraction. IEEE Trans Med Imag. (2020) 39:3619–29. doi: 10.1109/TMI.2020.3001036 PMC7665851 32746108

[40] Sun Y Dai D Zhang Q Wang Y Xu S Lian C. MSCA-Net: Multi-scale contextual attention network for skin lesion segmentation. Pattern Recognit. (2023) 139:109524. doi: 10.1016/j.patcog.2023.109524

[41] Liu Y Zhu Y Xin Y Zhang Y Yang D Xu T. MESTrans: Multi-scale embedding spatial transformer for medical image segmentation. Comput Methods Prog Biomed. (2023) 233:107493. doi: 10.1016/j.cmpb.2023.107493 36965298

[42] Heidari M Kazerouni A Soltany M Azad R Aghdam EK Cohen-Adad J. HiFormer: hierarchical multi-scale representations using transformers for medical image segmentation. In: 2023 IEEE/CVF Winter Conference on Applications of Computer Vision (WACV); 2023 Jan 3-7; Waikoloa, HI, USA: IEEE (2023). pp. 6191–201. doi: 10.1109/CVPR.2019.00953

[43] Yang Y Zhang L Ren L Wang X. MMVIT-Seg: A lightweight transformer and cnn fusion network for covid-19 segmentation. Comput Methods Prog Biomed. (2023) 230:107348. doi: 10.1109/WACV56688.2023.00614 PMC9833855 36706618

[44] Zhang Y Wang Y Zhang B Li Q. A hyperspectral dataset of precancerous lesions in gastric cancer and benchmarks for pathological diagnosis. J Biophoton. (2022) 15:e202200163. doi: 10.1002/jbio.202200163 35869783

